# Melatonin May Curtail the Metabolic Syndrome: Studies on Initial and Fully Established Fructose-Induced Metabolic Syndrome in Rats

**DOI:** 10.3390/ijms14022502

**Published:** 2013-01-25

**Authors:** Daniel P. Cardinali, Pablo A. Scacchi Bernasconi, Roxana Reynoso, Carlos F. Reyes Toso, Pablo Scacchi

**Affiliations:** 1 Department of Teaching and Research, Faculty of Medical Sciences, Pontificia Universidad Católica Argentina, 1107 Buenos Aires, Argentina; E-Mails: scacchipa@yahoo.com.ar (P.A.S.B.); rroxam@yahoo.com.ar (R.R.); pabloscacchi@gmail.com (P.S.); 2 Department of Physiology, Faculty of Medicine, University of Buenos Aires, 1121 Buenos Aires, Argentina; E-Mail: creyesto@fmed.uba.ar

**Keywords:** metabolic syndrome, melatonin, fructose, dyslipidemia, hypertension, obesity, glucose tolerance

## Abstract

To examine the effect of melatonin given to rats simultaneously with fructose on initial and fully developed metabolic syndrome, male Wistar rats had free access to chow and 5% or 10% fructose drinking solution for 8 weeks. As compared to controls, systolic blood pressure augmented significantly under both treatments whereas excessive body weight was seen in rats receiving the 10% fructose only. Rats drinking 5% fructose showed a greater tolerance to a glucose load while rats having access to a 10% fructose drinking solution exhibited the expected impaired glucose tolerance found in the metabolic syndrome. Circulating triglyceride and low density lipoproteins-cholesterol (LDL-c) concentration augmented significantly in rats showing a fully developed metabolic syndrome only, while high blood cholesterol levels were found at both stages examined. Melatonin (25 μg/mL drinking solution) counteracted the changes in body weight and systolic blood pressure found in rats administered with fructose. Melatonin decreased the abnormal hyperglycemia seen after a glucose load in 10% fructose-treated rats but it did not modify the greater tolerance to glucose observed in animals drinking 5% fructose. Melatonin also counteracted the changes in plasma LDL-c, triglyceride and cholesterol levels and decreased plasma uric acid levels. The results underline a possible therapeutical role of melatonin in the metabolic syndrome, both at initial and established phases.

## 1. Introduction

The metabolic syndrome is a cluster of metabolic abnormalities including, among others, abdominal obesity, insulin resistance, atherogenic dyslipidemia, increased blood pressure (BP) and a pro-inflammatory state [1–4]. Besides an increased risk for cardiovascular diseases and type 2 diabetes, this syndrome is associated with numerous co-morbidities including obstructive sleep apnea syndrome, reproductive disorders, dementia, non-alcoholic fatty liver disease and some cancers.

The metabolic syndrome is characterized by the presence of at least three of the following parameters: waist circumference >102 cm in males and >88 cm in females, triglycerides >150 mg/dL plasma, high density lipoproteins (HDL) <40 mg/dL plasma, BP > 130/85 mm Hg and fasting glucose >110 mg/dL [1–4]. The metabolic syndrome affects more that 25% of population in the developed and underdeveloped world with an associated threefold increased risk for cardiovascular mortality. It is therefore critical to identify mechanisms and strategies to prevent or treat it.

A causal role of dietary components has been postulated in the metabolic syndrome and fructose intake may play a major role in its etiology [4]. An impending increase in fructose intake, primarily in the form of sucrose (that contains 50% fructose) and corn syrup (55% fructose content) is documented in the last 25 years. Moreover, fructose intake has been linked to the increased incidence of obesity and diabetes [4].

High fructose intake has been commonly modeled in rats [5], and lately in non-human primates [6]. In both types of models, fructose feeding induces hyperinsulinemia, insulin resistance and hypertriglyceridemia. Additionally, hypertension is produced by feeding rats with a high-fructose diet [7].

Among several substances with the capacity to curtail fructose-induced metabolic syndrome, melatonin has received attention because of its very low or absent toxicity that turns it potentially appropriate for human use. In high-fat/high sucrose-fed rats giving an intraperitoneal (i.p.) injection of 4 mg/kg body weight melatonin every morning for 8 weeks, starting after 20 weeks of feeding, weight gain inhibition occurred together with improved insulin sensitivity [8]. Rats fed a diet containing 60% fructose exhibited an inhibition of melatonin secretion and turned hypertensive unless a daily supplementation of melatonin (30 mg/kg in drinking water) was given [9].

In a recent study, the melatonin activity on the metabolic syndrome induced by a diet containing 60% fructose was examined [10]. This diet increased serum insulin, triglyceride, total cholesterol, free fatty acids, uric acid, leptin and lipid peroxide concentrations as well as hepatic triglyceride and cholesterol concentrations. Insulin resistance, relative intra-abdominal fat and an augmented liver weight were also apparent. The daily i.p. administration of melatonin (1 or 10 mg/kg body weight), starting at 4 weeks of feeding, attenuated all these changes underlying the efficacy of melatonin to improve a fully developed metabolic syndrome [10].

The objective of the present study was to examine further the effect of melatonin given in the drinking solution simultaneously with fructose at two different stages of the induced metabolic syndrome: an initial stage, in which changes in BP and circulating lipids coexist with an augmented glucose tolerance and at the stage of the established metabolic syndrome, when insulin resistance and dyslipidemia fully develop. To attain this either a 5% or a 10% fructose drinking solution was given for 8 weeks.

## 2. Results and Discussion

### 2.1. Results

Chow consumption (g/rat/day) was similar for controls (16 ± 1), 5% fructose-fed rats (14 ± 1) and 10% fructose-fed rats (17 ± 2). Water consumption (ml/rat/day) was 25 ± 4 (controls), 36 ± 4 (5% fructose) and 41 ± 5 (10% fructose) (*F* = 3.53, *p* < 0.05, one way ANOVA, differences between control and 10% fructose groups being significant, Dunnett’s *t* test). Therefore, the individual total caloric intake (kcal/day was 46 ± 3 (controls), 55 ± 3 (5% fructose) and 59 ± 4 (*F* = 3.91, *p* < 0.04, one way ANOVA, differences between control and 10% fructose groups being significant, Dunnett’s *t* test). Chow or water consumption was not affected by melatonin. Table 1 summarizes the initial and final body weight, systolic BP and the changes in a number of blood analytes used clinically to assess the metabolic syndrome in the three groups of animals examined.

**Figure 1:**
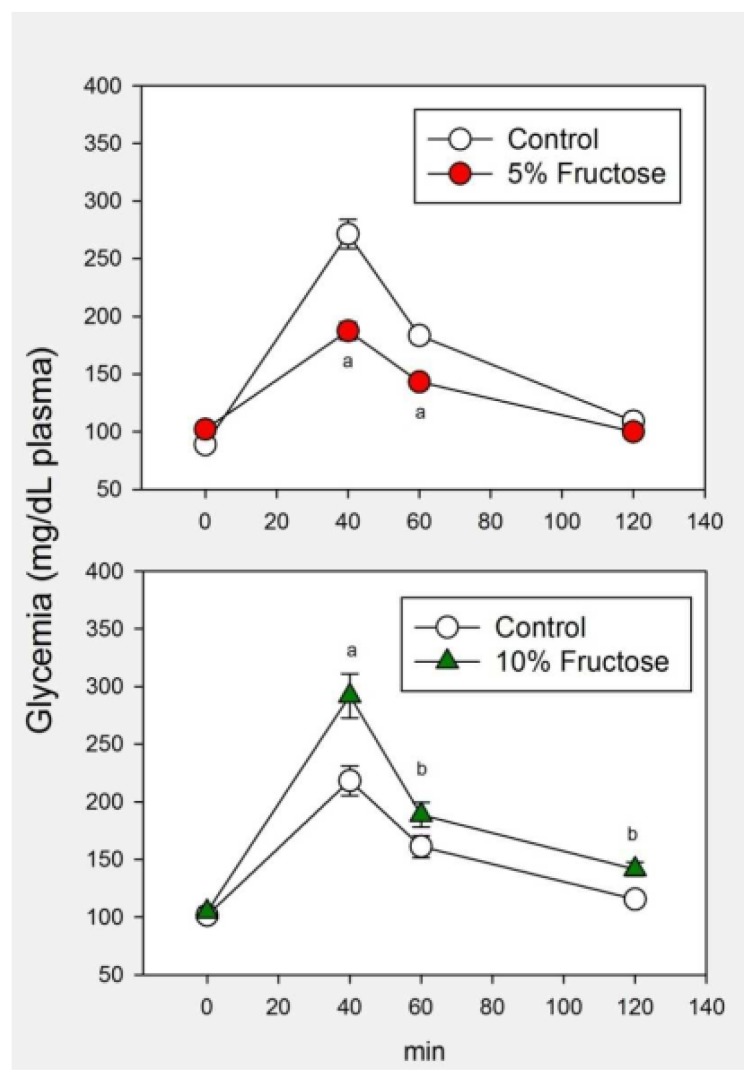
Glucose tolerance test in rats that had free access to chow and a drinking solution containing 5% fructose (upper panel) or 10% fructose (lower panel) for 8 weeks. Controls received tap water. Glucose (2 g/kg body weight) was administered i.p. Shown are the means ± SEM (*n* = 8 per group). Letters indicate the existence of significant differences *vs.* control (Student’s *t* test) ^a^*p* < 0.01, ^b^*p* < 0.03. For further statistical analysis see text.

As compared to controls, body weight augmented significantly in rats drinking the 10% fructose solution while systolic BP augmented significantly in both groups of fructose-overloaded rats. Rats drinking the 10% fructose solution showed significant increases in circulating low density lipoproteins (LDL)-c, cholesterol and triglyceride levels as compared to controls while only circulating cholesterol increased significantly in rats receiving 5% fructose. Blood levels of HDL-c, creatinine, urea and uric acid were indistinguishable from controls after fructose overload (Table 1). Figure 1 depicts the changes in circulating glucose levels in a glucose tolerance test in both experimental groups. When analyzed as a main factor in a factorial ANOVA the rats receiving the 5% fructose drinking solution exhibited significantly lower glycemia values after glucose administration than controls (*p* < 0.001). In contrast, the attained glycemia values in rats drinking a 10% fructose solution were significantly higher than in controls (*p* < 0.001).

**Figure 2:**
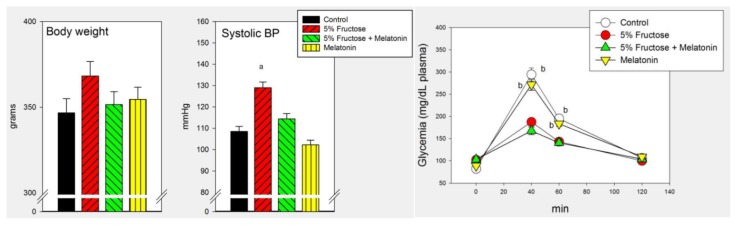
Body weight, systolic BP and glycemia after the administration of glucose (2 g/kg body weight i.p.) to rats that had free access to chow and drinking solutions containing 0.015% ethanol (control), 0.015% ethanol plus 5% fructose, 5% fructose plus 25 μg/mL melatonin or 25 μg/mL of melatonin for 8 weeks. Controls received tap water. Shown are the means ± SEM (*n* = 8 per group). Letters indicate the existence of significant differences between the experimental groups after a one-way ANOVA followed by a post-hoc Bonferroni’s test, ^a^*p* < 0.02 *vs.* the remaining groups, ^b^*p* < 0.02 *vs.* rats drinking 5% fructose.

**Figure 3:**
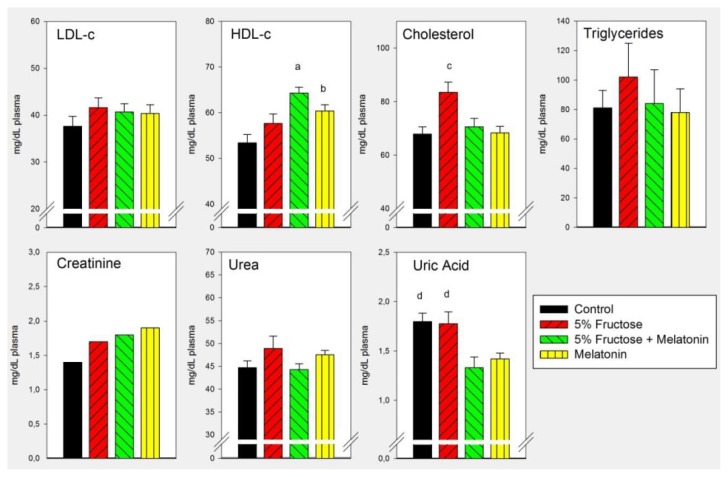
Plasma levels of LDL-c, HDL-c, cholesterol, triglycerides, creatinine, urea and uric acid in rats that had free access to chow and drinking solutions containing 0.015% ethanol (control), 0.015% ethanol plus 5% fructose, 5% fructose plus 25 μg/mL of melatonin or 25 μg/mL of melatonin for 8 weeks. Controls received tap water. Shown are the means ± SEM (*n* = 8 per group). Letters indicate the existence of significant differences between the experimental groups after a one-way ANOVA followed by a post-hoc Bonferroni’s test, ^a^*p* < 0.01 *vs.* control; ^b^*p* < 0.05 *vs.* control; ^c^*p* < 0.02 *vs.* the remaining groups; ^d^*p* < 0.04 *vs*. melatonin-treated rats. For further statistical analysis, see text.

The efficacy of the concomitant administration of melatonin to overcome the metabolic changes brought about by a 5% fructose drinking solution is depicted in Figure 2 and Figure 3. Melatonin counteracted significantly the changes in systolic BP in rats at this early stage of the metabolic syndrome but failed to affect the increased glucose tolerance observed (Figure 2).

As shown in Figure 3, melatonin counteracted the changes in plasma cholesterol found in rats at this early stage of the metabolic syndrome. When analyzed as main factors in the factorial ANOVA melatonin decreased plasma uric acid levels (*p* < 0.001).

**Figure 4:**
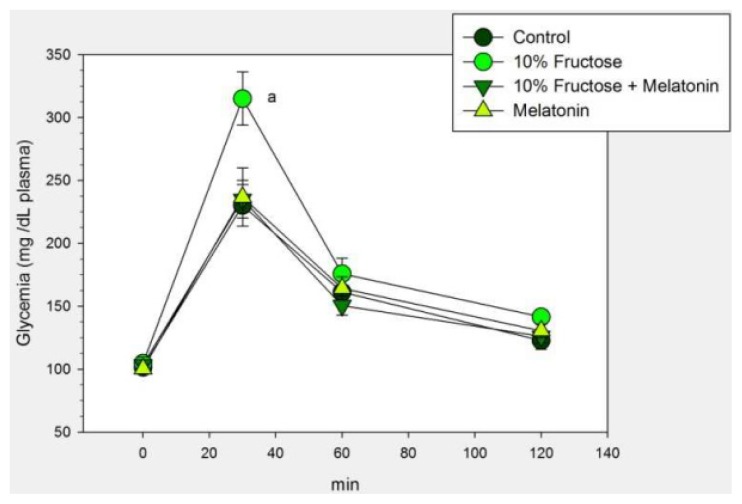
Glucose tolerance test in rats that had free access to chow and drinking solutions containing 0.015% ethanol (control), 0.015% ethanol plus 10% fructose, 10% fructose plus 25 μg/mL of melatonin or 25 μg/mL of melatonin for 8 weeks. Controls received tap water. A glucose solution of 2 g/kg body weight was administered i.p. Shown are the means ± SEM (*n* = 8 per group). ^a^*p* < 0.02 *vs*. the remaining groups, one-way ANOVA, Bonferroni’s test.

Table 2 and Figure 4 summarized the effect of the concomitant administration of melatonin on the changes caused by a 10% fructose drinking solution in rats. Melatonin counteracted significantly body weight and systolic BP at this established phase of the metabolic syndrome (Table 2). Melatonin also counteracted the decreased glucose tolerance found in 10% fructose-fed rats as demonstrated by the lower glycemia values attained after i.p. glucose administration (Figure 4). In addition, melatonin treatment counteracted significantly the increase in LDL-c, triglyceride and cholesterol concentration (Table 2). Analyzed as main factor in the factorial ANOVA melatonin depressed plasma uric acid (*p* < 0.001) at this established phase of the metabolic syndrome (Table 2).

### 2.2. Discussion

A high fructose (50%–60%) solid diet in male rats induces metabolic alterations similar to those found in metabolic syndrome, including insulin resistance and hypertension [5]. However, feeding diets incorporating fructose in drinking water (10% *w*/*v*) for 2 weeks induce in male rats hypertriglyceridemia and fatty liver without modifying or even increasing plasma glucose tolerance to a glucose load [11,12].

We took advantage of this approach to define two stages of the metabolic syndrome caused by fructose, by giving for 8 weeks either a 5% or a 10% fructose solution (in which fructose accounted for 21%–27% and 48%–57% of total caloric intake, respectively [13]). Rats receiving 5% fructose exhibited a greater tolerance to glucose, as demonstrated by the lower glycemia values achieved after i.p. glucose administration as compared to controls. In contrast, rats having access to a 10% fructose drinking solution showed an impaired glucose tolerance compatible with insulin resistance.

As compared to controls, systolic BP augmented significantly in both experimental groups whereas significant body weight changes were seen in rats receiving the 10% fructose solution only. Blood cholesterol levels augmented significantly in both groups of animals while circulating triglyceride and LDL-c concentration augmented significantly in rats receiving 10% fructose only. Hence, two different stages of a metabolic syndrome brought about by fructose could be defined: an initial stage, in which changes in BP and circulating lipids coexist with an augmented glucose tolerance, and an established stage, when a decreased glucose tolerance and circulating lipid changes were fully developed.

A number of studies indicate that melatonin has the ability to reduce type 2 diabetes and liver steatosis [14,15]. In addition, melatonin treatment induces regeneration/proliferation of β-cells in pancreas which leads to a decrement in blood glucose in streptozotocin-induced type 1 diabetic rats [16]. Loss of circulating melatonin via pinealectomy results in marked hyperinsulinemia and accumulation of triglycerides in the liver [17].

Long-term administration of melatonin improves lipid metabolism in type 2 diabetic rats through amelioration of insulin resistance [18]. In the present study melatonin decreased the high levels of glucose caused by a glucose load in 10% fructose-treated rats only. It also counteracted the increase in body weight found in rats with fully developed metabolic syndrome. These results fit with previous observations indicating that melatonin can effectively reduce adiposity in rats giving a high fructose diet [8–10] as well as in other models of hyperadiposity [19–26]. A remarkable observation in most of these studies is that the decrease in body weight after administering melatonin occurred in the absence of significant differences in food intake. A key piece of evidence in this respect is the observation that melatonin plays a fundamental role in the seasonal changes of adiposity of Siberian hamsters by increasing the activity of the sympathetic nervous system innervating white fat, thereby increasing lipolysis [27]. Whether or not a similar mechanism is also operative in a non-seasonal species like the laboratory rat remains to be defined. Alternatively, the weight-loss-promoting effect of melatonin may be attributable to an increase in energy expenditure by brown adipose tissue [28].

Collectively, the present and previous results indicate that the administration of melatonin effectively counteracts some of the disrupting effects seen in diet-induced obesity in rats, in particular, insulin resistance, dyslipidemia and overweight. It should be noted that there is a critical need for studies on melatonin effects on the metabolic syndrome phenotype in primates, since all animal studies demonstrating effects of melatonin on metabolism have been conducted in nocturnal species.

In accordance with previous observations [9], melatonin was also effective to decrease the augmented BP found in rats drinking either a 5% or a 10% fructose solution. The present study describes for the first time that the effect of melatonin can be seen at an early phase of the metabolic syndrome, thus underlying its potentiality in preventing and treating the syndrome. Indeed, nighttime melatonin supplementation reduced nocturnal BP in otherwise untreated hypertensive men [29], nondipping women [30], patients with nocturnal hypertension [31] and in adolescents with type 1 diabetes mellitus [32].

Melatonin, while counteracting the changes in plasma LDL-c, triglyceride and cholesterol, decreased plasma uric acid levels. This last effect can be of potential therapeutic value in view that hyperuricemia has a pathogenic role in metabolic syndrome, possibly due to its ability to inhibit endothelial function [33].

There is considerable evidence that circadian misalignment is associated with increased risk of obesity, diabetes, and cardiovascular disease [34]. Life style changes, such as nocturnality and overly rich diets, are followed by disruption of the sleep/wake cycle and other circadian rhythms. Due to its effects on circadian rhythmicity melatonin can provide the basis for a therapeutic strategy in metabolic syndrome. A consensus of the British Association for Psychopharmacology on evidence-based treatment of insomnia, parasomnia and circadian rhythm sleep disorders concluded that melatonin is the first choice treatment when a hypnotic is indicated in patients over 55 years [35].

Since melatonin has a short half life (less than 30 min) a number of melatonin agonists with a longer duration of action have been developed. Concerning their potential to be used therapeutically in the metabolic syndrome, ramelteon (Rozerem^®^, Takeda Pharmaceuticals, Kyoto, Japan) attenuated age-associated hypertension and weight gain in spontaneously hypertensive rats [36], agomelatine (Valdoxan^®^, Servier, Neuilly-sur-Seine, France) reduced seasonal body weight increase in rodents [37] and Neu-P11 (piromelatine) improved insulin sensitivity in a rodent model of metabolic syndrome [8]. The doses and the relative potencies of the melatonin agonists employed indicate that the regular melatonin dose to treat insomnia (2–5 mg melatonin/day) is probably unsuitable to protect against several comorbilities of the metabolic syndrome. Indeed, diabetes and concomitant oxyradical-mediated damage, inflammation, microvascular disease and atherothrombotic risk are effectively prevented by high doses of melatonin in a number of animal models [38,39]. If one expects melatonin to be an effective cytoprotector it is likely that the low doses of melatonin employed so far are not very beneficial.

## 3. Experimental Section

### 3.1. Animals and Experimental Design

Male Wistar rats (60 days of age) were kept under standard conditions of controlled light (12:12 h light/dark schedule; lights on at 08:00 h) and temperature (22 ± 2 °C). Three experiments were performed.

In experiment 1, groups of 8 rats had free access for 8 weeks to chow and one of the following drinking solutions: (i) a 5% fructose solution (in which fructose accounted for 21%–27% of caloric intake [13]); (ii) a 10% fructose solution (in which fructose accounted for 48%–57% of caloric intake [13]); (iii) tap water. Rat chow contained 60% carbohydrate mainly as starch with less than 0.4% fructose.

In experiment 2, rats were randomly divided into four groups (*n* = 8 per group) and had free access to chow and one of the following drinking solutions for 8 weeks: (i) 5% fructose; (ii) 5% fructose plus 25 μg/mL of melatonin; (iii) 25 μg/mL of melatonin; (iv) tap water. Since ethanol was used as a melatonin’s vehicle, drinking solutions in groups (i) and (iv) were added 0.015% ethanol. Experiment 3 had a similar design, except for that a 10% fructose solution was tested.

Chow and water consumption were measured weekly. Caloric intake for fructose-fed rats was calculated as sum of calories ingested as food on the basis of 2.9 kcal per gram of chow consumed and on that each ingested gram of fructose corresponds to 4.0 kcal.

The daily melatonin dosage used was 1.9–3.2 mg/kg. The human equivalence dose, calculated by using the body surface area normalization method [40] is 0.31–0.52 mg/kg (*i.e.*, 21–35 mg/day for a 70 kg adult).

### 3.2. BP Measurement

Systolic BP was measured by using a manometer-tachometer (Rat Tail NIBP System; ADInstruments Pty Ltd., Sydney, Australia) employing an inflatable tail-cuff connected to a MLT844 Physiological Pressure Transducer (ADInstruments) and PowerLab data acquisition unit (ADInstruments). Rats were placed in a plastic holder mounted on a thermostatically controlled warm plate that was maintained at 35 °C during measurements. An average value from three BP readings (that differed by no more than 2 mm Hg) was determined for each animal after they became acclimated to the environment. All BP measurements were made between 09:00 and 12:00 h.

### 3.3. Biochemical Assays

A glucose tolerance test was performed at 09:00 h after a 2-h fast. Rats were anesthetized, and following the collection of an unchallenged sample (time 0), a glucose solution of 2 g/kg body weight was administered i.p. During the test, blood was collected from the saphenous vein at 30, 60 and 120 min after glucose administration to measure glucose concentration.

The rats were then eutanized by decapitation under conditions of minimal stress. All experiments were conducted in accordance with the guidelines of the International Council for Laboratory Animal Science (ICLAS). Trunk blood was collected and plasma samples were obtained by centrifugation of blood at 1500× *g* for 15 min. EDTA (6 g/100 mL) was used as an anticoagulant. Samples were stored at −70 °C until further analysis.

Glycemia was measured using the Accu-Check Compact kit (Roche Diagnostics, Indianapolis, IN, USA). The plasma lipid profile was determined by measuring the content of triglycerides, total cholesterol, HDL-c and LDL-c using commercially available reagent kits as per the manufacturer’s instructions (BioSystems S.A., Buenos Aires, Argentina). Creatinine, urea and uric acid were measured by standard enzymatic procedures (BioSystems S.A.).

### 3.4. Statistical Analysis

After verifying normality of distribution of data, the statistical analysis of the results was performed by a one-way or a two-way factorial analysis of variance (ANOVA) followed by Bonferroni’s multiple comparison, Dunnett’s or Student’s *t* tests, as stated. *p* values lower than 0.05 were taken as evidence of statistical significance.

## 4. Conclusions

Although understanding of the melatonin’s action in the pathogenesis of the metabolic syndrome is yet inconclusive, studies so far points out that melatonin through its chronobiotic, immunomodulatory, antioxidant and antiapoptotic actions can exert beneficial effects on the metabolic syndrome phenotype. The present study in fructose-treated rats with an initial or fully developed metabolic syndrome underlines a possible therapeutical role of melatonin in the metabolic syndrome, both at initial and established phases. The results support the concept that melatonin can be a useful add-on therapy to curtail insulin resistance, dyslipidemia and overweight in obese individuals [15].

## Figures and Tables

**Table 1:** Body weight, systolic BP and plasma levels of several analytes in rats receiving a 5% or a 10% fructose overload for 8 weeks.

	Control	5% Fructose	10% Fructose	*F*	*p*
Initial body weight (g)	273 ± 15	267 ± 9	269 ± 11	0.07	NS
Final body weight (g)	354 ± 13	339 ± 11	409 ± 19	6.26	0.007
Systolic BP (mmHg)	108 ± 4	124 ± 5 *	129 ± 5 *	5.47	0.012
LDL-c (mg/dL)	37 ± 5	45 ± 4	54 ± 4 *	3.81	0.039
HDL-c (mg/dL)	53 ± 2	52 ± 1	54 ± 4	0.14	NS
Cholesterol (mg/dL)	68 ± 4	84 ± 4 *	88 ± 6 *	4.94	0.017
Triglycerides (mg/dL)	95 ± 6	112 ± 11	233 ± 19 *	32.8	<0.001
Creatinine (mg/dL)	1.1 ± 0.1	1.2 ± 0.2	1.1 ± 0.1	1.11	NS
Urea (mg/dL)	44 ± 5	49 ± 6	39 ± 3	0.35	NS
Uric acid (mg/dL)	1.8 ± 0.2	1.7 ± 0.2	1.6 ± 0.3	0.18	NS

Shown are the means ± SEM (*n* = 8 per group). *F* values in ANOVA and the corresponding *p* are quoted. NS: not significant. Asterisks designate the existence of significant differences *vs*. control in a one-way ANOVA followed by a Dunnett’s *t* test.

**Table 2:** Effect of melatonin on body weight, systolic BP and plasma levels of several analytes in rats with a fully developed metabolic syndrome (10% fructose drinking solution for 8 weeks).

	Control	10% Fructose	10% Fructose + Melatonin	Melatonin	*F*	*p*
Initial body weight (g)	265 ± 22	284 ± 19	273 ± 16	269 ± 26	0.15	NS
Final body weight (g)	351 ± 30	479 ± 36 a	370 ± 32	371 ± 30	3.29	0.035
Systolic BP (mmHg)	102 ± 8	129 ± 6 a	103 ± 4	100 ± 8 b	4.18	0.014
LDL-c (mg/dL)	39 ± 4	59 ± 6 c	19 ± 3 d	22 ± 4	17.6	<0.001
HDL-c (mg/dL)	54 ± 7	55 ± 8	62 ± 6	58 ± 5	0.29	NS
Cholesterol (mg/dL)	65 ± 6	88 ± 4 e	67 ± 5	71 ± 4	4.71	0.009
Triglycerides (mg/dL)	175 ± 23	302 ± 26 f	215 ± 19 g	164 ± 13	9.04	<0.001
Creatinine (mg/dL)	1.1 ± 0.1	1.2 ± 0.2	1.1 ± 0.1	1.3 ± 0.1	0.24	NS
Urea (mg/dL)	44 ± 5	40 ± 6	38 ± 3	42 ± 4	0.31	NS
Uric acid (mg/dL)	1.7 ± 0.1	1.9 ± 0.2 h	1.2 ± 0.1	1.1 ± 0.1 d	8.52	<0.001

For experimental details see Methods. Shown are the means ± SEM (*n* = 8 per group). Letters indicate the existence of significant differences between the experimental groups after a one-way ANOVA followed by a post-hoc Bonferroni’s test, as follows:

a *p* < 0.05 *vs*. control;

b *p* < 0.03 *vs*. fructose;

c *p* < 0.01 *vs*. the remaining groups;

d *p* < 0.02 *vs*. control;

e *p* < 0.02 *vs*. control and fructose + melatonin groups;

f *p* < 0.01 *vs*. control and melatonin alone groups;

g *p* < 0.04 *vs*. fructose;

h *p* < 0.01 *vs*. fructose + melatonin and melatonin groups. For further statistical analysis, see text.

## References

[1] Brown T., Avenell A., Edmunds L.D., Moore H., Whittaker V., Avery L., Summerbell C. (2009). Systematic review of long-term lifestyle interventions to prevent weight gain and morbidity in adults. Obes. Rev.

[2] Garaulet M., Madrid J.A. (2009). Chronobiology, genetics and metabolic syndrome. Curr. Opin. Lipidol.

[3] Maury E., Ramsey K.M., Bass J. (2010). Circadian rhythms and metabolic syndrome: From experimental genetics to human disease. Circ. Res.

[4] Tappy L., Le K.A., Tran C., Paquot N. (2010). Fructose and metabolic diseases: New findings, new questions. Nutrition.

[5] Tran L.T., Yuen V.G., McNeill J.H. (2009). The fructose-fed rat: A review on the mechanisms of fructose-induced insulin resistance and hypertension. Mol. Cell. Biochem.

[6] Bremer A.A., Stanhope K.L., Graham J.L., Cummings B.P., Wang W., Saville B.R., Havel P.J. (2011). Fructose-fed rhesus monkeys: A nonhuman primate model of insulin resistance, metabolic syndrome, and type 2 diabetes. Clin. Transl. Sci.

[7] Shimamoto K., Ura N. (2006). Mechanisms of insulin resistance in hypertensive rats. Clin. Exp. Hypertens.

[8] She M., Deng X., Guo Z., Laudon M., Hu Z., Liao D., Hu X., Luo Y., Shen Q., Su Z., Yin W. (2009). NEU-P11, a novel melatonin agonist, inhibits weight gain and improves insulin sensitivity in high-fat/high-sucrose-fed rats. Pharmacol. Res.

[9] Leibowitz A., Peleg E., Sharabi Y., Shabtai Z., Shamiss A., Grossman E. (2008). The role of melatonin in the pathogenesis of hypertension in rats with metabolic syndrome. Am. J. Hypertens.

[10] Kitagawa A., Ohta Y., Ohashi K. (2012). Melatonin improves metabolic syndrome induced by high fructose intake in rats. J. Pineal Res.

[11] Roglans N., Sanguino E., Peris C., Alegret M., Vazquez M., Adzet T., Diaz C., Hernandez G., Laguna J.C., Sanchez R.M. (2002). Atorvastatin treatment induced peroxisome proliferator-activated receptor alpha expression and decreased plasma nonesterified fatty acids and liver triglyceride in fructose-fed rats. J. Pharmacol. Exp. Ther.

[12] Park J., Lemieux S., Lewis G.F., Kuksis A., Steiner G. (1997). Chronic exogenous insulin and chronic carbohydrate supplementation increase *de novo* VLDL triglyceride fatty acid production in rats. J. Lipid Res.

[13] Dai S., McNeill J.H. (1995). Fructose-induced hypertension in rats is concentration- and duration-dependent. J. Pharmacol. Toxicol. Methods.

[14] Peschke E., Stumpf I., Bazwinsky I., Litvak L., Dralle H., Muhlbauer E. (2007). Melatonin and type 2 diabetes—A possible link?. J. Pineal Res.

[15] Nduhirabandi F., Du Toit E.F., Lochner A. (2012). Melatonin and the metabolic syndrome: A tool for effective therapy in obesity-associated abnormalities?. Acta Physiol.

[16] Kanter M., Uysal H., Karaca T., Sagmanligil H.O. (2006). Depression of glucose levels and partial restoration of pancreatic beta-cell damage by melatonin in streptozotocin-induced diabetic rats. Arch. Toxicol.

[17] Nishida S., Sato R., Murai I., Nakagawa S. (2003). Effect of pinealectomy on plasma levels of insulin and leptin and on hepatic lipids in type 2 diabetic rats. J. Pineal Res.

[18] Nishida S., Segawa T., Murai I., Nakagawa S. (2002). Long-term melatonin administration reduces hyperinsulinemia and improves the altered fatty-acid compositions in type 2 diabetic rats via the restoration of Delta-5 desaturase activity. J. Pineal Res.

[19] Prunet-Marcassus B., Desbazeille M., Bros A., Louche K., Delagrange P., Renard P., Casteilla L., Penicaud L. (2003). Melatonin reduces body weight gain in Sprague Dawley rats with diet-induced obesity. Endocrinology.

[20] Puchalski S.S., Green J.N., Rasmussen D.D. (2003). Melatonin effect on rat body weight regulation in response to high-fat diet at middle age. Endocrine.

[21] Sartori C., Dessen P., Mathieu C., Monney A., Bloch J., Nicod P., Scherrer U., Duplain H. (2009). Melatonin improves glucose homeostasis and endothelial vascular function in high-fat diet-fed insulin-resistant mice. Endocrinology.

[22] Rios-Lugo M.J., Cano P., Jimenez-Ortega V., Fernandez-Mateos M.P., Scacchi P.A., Cardinali D.P., Esquifino A.I. (2010). Melatonin effect on plasma adiponectin, leptin, insulin, glucose, triglycerides and cholesterol in normal and high fat-fed rats. J. Pineal Res.

[23] Ladizesky M.G., Boggio V., Albornoz L.E., Castrillón P., Mautalen C.A., Cardinali D.P. (2003). Melatonin increases oestradiol-induced bone formation in ovariectomized rats. J. Pineal Res.

[24] Sanchez-Mateos S., Alonso-Gonzalez C., Gonzalez A., Martinez-Campa C.M., Mediavilla M.D., Cos S., Sanchez-Barcelo E.J. (2007). Melatonin and estradiol effects on food intake, body weight, and leptin in ovariectomized rats. Maturitas.

[25] Hussein M.R., Ahmed O.G., Hassan A.F., Ahmed M.A. (2007). Intake of melatonin is associated with amelioration of physiological changes, both metabolic and morphological pathologies associated with obesity: an animal model. Int. J. Exp. Pathol.

[26] Raskind M.A., Burke B.L., Crites N.J., Tapp A.M., Rasmussen D.D. (2007). Olanzapine-induced weight gain and increased visceral adiposity is blocked by melatonin replacement therapy in rats. Neuropsychopharmacology.

[27] Bartness T.J., Demas G.E., Song C.K. (2002). Seasonal changes in adiposity: The roles of the photoperiod, melatonin and other hormones, and sympathetic nervous system. Exp. Biol. Med.

[28] Tan D.X., Manchester L.C., Fuentes-Broto L., Paredes S.D., Reiter R.J. (2011). Significance and application of melatonin in the regulation of brown adipose tissue metabolism: Relation to human obesity. Obes. Rev.

[29] Scheer F.A., van Montfrans G.A., van Someren E.J., Mairuhu G., Buijs R.M. (2004). Daily nighttime melatonin reduces blood pressure in male patients with essential hypertension. Hypertension.

[30] Cagnacci A., Cannoletta M., Renzi A., Baldassari F., Arangino S., Volpe A. (2005). Prolonged melatonin administration decreases nocturnal blood pressure in women. Am. J. Hypertens.

[31] Grossman E., Laudon M., Yalcin R., Zengil H., Peleg E., Sharabi Y., Kamari Y., Shen-Orr Z., Zisapel N. (2006). Melatonin reduces night blood pressure in patients with nocturnal hypertension. Am. J. Med.

[32] Cavallo A., Daniels S.R., Dolan L.M., Bean J.A., Khoury J.C. (2004). Blood pressure-lowering effect of melatonin in type 1 diabetes. J. Pineal Res.

[33] Nakagawa T., Hu H., Zharikov S., Tuttle K.R., Short R.A., Glushakova O., Ouyang X., Feig D.I., Block E.R., Herrera-Acosta J. (2006). A causal role for uric acid in fructose-induced metabolic syndrome. Am. J. Physiol. Renal Physiol.

[34] Scheer F.A., Hilton M.F., Mantzoros C.S., Shea S.A. (2009). Adverse metabolic and cardiovascular consequences of circadian misalignment. Proc. Natl. Acad. Sci. USA.

[35] Wilson S.J., Nutt D.J., Alford C., Argyropoulos S.V., Baldwin D.S., Bateson A.N., Britton T.C., Crowe C., Dijk D.J., Espie C.A. (2010). British association for psychopharmacology consensus statement on evidence-based treatment of insomnia, parasomnias and circadian rhythm disorders. J. Psychopharmacol.

[36] Oxenkrug G.F., Summergrad P. (2010). Ramelteon attenuates age-associated hypertension and weight gain in spontaneously hypertensive rats. Ann. N. Y. Acad. Sci.

[37] Guardiola-Lemaitre B. (2007). Melatoninergic receptor agonists and antagonists: Therapeutic perspectives. J. Soc. Biol.

[38] Cardinali D.P., Cano P., Jimenez-Ortega V., Esquifino A.I. (2011). Melatonin and the metabolic syndrome: Physiopathologic and therapeutical implications. Neuroendocrinology.

[39] Hardeland R., Cardinali D.P., Srinivasan V., Spence D.W., Brown G.M., Pandi-Perumal S.R. (2011). Melatonin—A pleiotropic, orchestrating regulator molecule. Prog. Neurobiol.

[40] Reagan-Shaw S., Nihal M., Ahmad N. (2007). Dose translation from animal to human studies revisited. FASEB J.

